# Inhibitory dysfunction may cause prospective memory impairment in temporal lobe epilepsy (TLE) patients: an event-related potential study

**DOI:** 10.3389/fnhum.2023.1006744

**Published:** 2023-07-26

**Authors:** Hemei Yu, Junling Gao, Richard Shek-Kwan Chang, Windsor Mak, Thuan-Quoc Thach, Raymond Tak Fai Cheung

**Affiliations:** ^1^Department of Medicine, School of Clinical Medicine, Li Ka Shing Faculty of Medicine, The University of Hong Kong, Hong Kong, Hong Kong SAR, China; ^2^Centre of Buddhist Studies, The University of Hong Kong, Hong Kong, Hong Kong SAR, China; ^3^Division of Neurology, Department of Medicine, Queen Mary Hospital, Hong Kong, Hong Kong SAR, China; ^4^Department of Psychiatry, School of Clinical Medicine, Li Ka Shing Faculty of Medicine, The University of Hong Kong, Hong Kong, Hong Kong SAR, China

**Keywords:** temporal lobe epilepsy (TLE), prospective memory (PM), working memory, inhibition, event-related potential (ERP), Go/Nogo, Oddball

## Abstract

**Introduction:**

Prospective memory (PM) is the ability to remember future intentions, and PM function is closely related to independence in daily life, particularly in patients with temporal lobe epilepsy (TLE). As PM involves various cognitive components of attention, working memory, inhibition and other executive functions, this study investigated how TLE may affect PM components and the underlying neural mechanisms.

**Methods:**

Sixty-four subjects were recruited, including 20 refractory TLE patients, 18 well-controlled TLE patients and 26 age-matched healthy controls. A set of neuropsychological tests was administered to assess specific brain functions. An event-related potential (ERP) task was used to further explore how PM and its components would be differentially affected in the two TLE types.

**Results:**

Our findings revealed that: (1) refractory TLE patients scored lower than the healthy controls in the digit span, Verbal Fluency Test and Symbol Digit Modalities Test; (2) refractory TLE patients exhibited impaired PM performance and reduced prospective positivity amplitudes over the frontal, central and parietal regions in ERP experiments when compared to the healthy controls; and (3) decreased P3 amplitudes in the nogo trials were observed over the frontal-central sites in refractory but not in well-controlled TLE patients.

**Discussion:**

To our knowledge, this is the first ERP study on PM that has specifically identified PM impairment in refractory but not in well-controlled TLE patients. Our finding of double dissociation in PM components suggests that inhibition dysfunction may be the main reason for PM deficit in refractory TLE patients. The present results have clinical implications for neuropsychological rehabilitation in TLE patients.

## Highlights

- Refractory temporal lobe epilepsy (TLE) patients performed worse than healthy controls in neuropsychological tests.
- Prospective positivity amplitudes were lower in refractory TLE patients and correlated with their impaired prospective memory (PM) function.
- Impairment in inhibition function may be the main reason for worse PM in refractory TLE patients.

## 1. Introduction

Temporal lobe epilepsy (TLE) is the most common type of epilepsy. TLE patients can have frequent seizures and cognitive dysfunction. The latter may severely compromise TLE patients’ daily functirons and social life (Black et al., 2010). Prospective memory (PM) performance relies on a set of cognitive abilities to enable us to maintain an intention for future actions by responding to event- or time-based cues which, in turn, trigger the intended action at the appropriate time (Kourtesis et al., 2021). Numerous studies in TLE patients have shown cognitive impairment, particularly in memory and executive functions (Stafstrom, 2007; Jackson-Tarlton et al., 2020; Kloc et al., 2022). As these functions are essential for PM performance, it is plausible that TLE patients have PM impairment.

Several studies in epilepsy patients have reported poor PM performance alongside memory and executive dysfunction (Adda et al., 2008; Wandschneider et al., 2010; Rai et al., 2015; Mills et al., 2022). For instance, Adda et al. (2008) reported that PM impairment in patients with mesial TLE is associated with hippocampal sclerosis, suggesting a significant role of the hippocampus in PM. PM may be impaired in other epilepsy syndromes. For example, PM impairment in patients with juvenile myoclonic epilepsy is genetically determined (Wandschneider et al., 2009, 2010). Despite these findings, there is a scarcity of neurophysiological research focused on PM in TLE patients, particularly concerning the underlying neural basis. This underscores the need for further investigation into the neural mechanisms of PM impairment in TLE and other epilepsy syndromes.

To successfully perform a PM task, an individual must execute a planned action upon encountering an external or internal cue. Paying attention to the cue is necessary for PM performance. In addition, the ongoing tasks must be ceased to allow for accomplishment of the PM task (Graf and Uttl, 2001). Another critical component of PM is memory itself, which is called the retrospective memory component in PM tasks. Memory impairment can cause PM failure. Our previous studies have found that patients with dementia and healthy elderly individuals exhibit varying degrees of PM impairment with long- and short-range brain fasciculi potentially playing differential roles (Gao et al., 2013, 2014). Innumerable neural fasciculi contribute to the dynamic function of the brain networks.

Similar to dementia and schizophrenia, epilepsy is also considered a neural network disease affecting not only local brain regions such as the temporal lobe but also other more distant brain areas (Kanner et al., 2017; Scharfman et al., 2018). Such network dysfunction may impact various cognitive functions in epilepsy patients, including attention, working memory, inhibition and execution. Several studies, together with a review, have reported evidence of working memory dysfunction in TLE patients (Wagner et al., 2009; Stretton and Thompson, 2012; Stretton et al., 2013). As the brain network responsible for executive function overlaps with that for working memory function, working memory deficits in TLE patients may lead to executive dysfunction.

Numerous studies have reported pathological brain network activity that may cause memory impairment in epilepsy (Kucewicz et al., 2013), and these altered brain networks could induce other cognitive dysfunction in TLE patients (Yang et al., 2018). Memory impairment is well-known in epilepsy (Hall et al., 2009; Parra-Diaz and Garcia-Casares, 2019). However, fewer studies in epilepsy patients have focused on PM performance. Investigating PM performance in TLE patients is essential because PM impairment adversely affects short-term tasks, long-term episodic tasks, and repetitive routine activities. Adequate PM function is crucial for the independence of all patients with any neurological disease in daily activities such as taking medication after meal and turning off the stove on time.

Recent studies on cognitive profile have provided valuable insights into the neuropsychological deficits and neural correlates in TLE (Hermann and Seidenberg, 2007; Reyes et al., 2019). Furthermore, a recent study has documented a large scale disorganization of memory and executive function in adult TLE patients (Caciagli et al., 2023); these findings corroborated with results in pediatric TLE patients (Oyegbile et al., 2018). Taken together, these studies highlight our evolving understanding of cognitive impairments in TLE and the importance of further investigation into the underlying neural mechanisms.

Neuropsychological questionnaires have been used to assess executive and other cognitive functions in TLE patients, including the Faux Pas test (Black et al., 2010), Wisconsin card sorting task (Ma et al., 2007), Stroop test (Labudda et al., 2009), Trail-making test (Ma et al., 2007), and Delis-Kaplan executive function system test (Takaya et al., 2006). These questionnaires could help researcher evaluate and understand PM function. However, questionnaires typically cannot differentiate among neural correlates such as attention, working memory, inhibition and execution, which are required for PM performance.

Executive function also plays a significant role in PM performance. Taxing the central executive processes of working memory can reduce the efficiency of PM (West et al., 2007). The executive system facilitates PM by tuning the responsiveness of neural systems within the extrastriate, posterior temporal, and frontal association cortices that support performance processing in PM (McNerney, 2006; West et al., 2007). It has been reported that PM performance requires several cognitive processes, mediated by brain regions including the subcortical-frontal-parietal network and limbic-hippocampal memory network (Adda et al., 2008; Burgess et al., 2011; Alves et al., 2019). Regarding the two most specific functions, attention allocation and ongoing task inhibition, however, their relevant neural mechanisms have not yet been clarified. Clarification of these mechanisms can permit specific treatment or clinical application.

This study aimed to use the event-related potential (ERP) technique to investigate attention and inhibition in PM. ERP offers a high temporal resolution and can help delineate potential impairments through typical neuropsychological paradigms like the Oddball experiment and Go/Nogo experiment (Adda et al., 2008; Burgess et al., 2011; Alves et al., 2019). To delineate the neural mechanism of PM and prospective positivity, this study also examined the roles of attention/working memory and inhibition in PM using behavioral and ERP methods. Specifically, we aimed to differentiate between these key components in PM performance of TLE patients using a series of ERP experiments. We hypothesized that TLE patients might exhibit worse PM performance than healthy controls and that PM impairment might differ between refractory and well-controlled TLE patients. This distinction can only be made through further investigation into the different components of PM.

## 2. Experimental procedures

### 2.1. Participants

Adult patients aged 61 years or younger and having a clinical diagnosis of TLE with or without secondary generalization were recruited from the Epilepsy Clinic of Queen Mary Hospital, Hong Kong. They were divided into the refractory (REF) group and well-controlled (WEL) group. TLE diagnosis was based on medical history, seizure semiology, MRI findings and multiple EEG recordings in accordance with the International League against Epilepsy guidelines (Reynolds, 2002). All patients have a temporal lobe lesion on MRI and/or temporal lobe epileptiform discharges on EEG and/or other evidence of TLE. REF group of patients had experienced three or more complex partial epileptic seizures per half-year in the preceding 1-year period despite using at least three antiepileptic drugs. WEL group of patients had had no epileptic seizures within the same period. All the patients have a TLE history of at least 3 years. There was no significant difference between the two epilepsy groups concerning the age of epilepsy onset using an independent *t*-test. However, the REF group had a significantly longer duration of epilepsy. Patients with other neurological or psychiatric diseases were excluded. Healthy age- and sex-matched native Cantonese-speaking controls with no neurological or psychiatric history were also recruited as the healthy (HEA) group. Becker’s Depression Inventory (BDI) was used to exclude participants with severe depression (Steer et al., 1999). Only right-handed subjects were included. The study protocol was approved by the Institutional Review Board of the University of Hong Kong/Hospital Authority Hong Kong West Cluster for human research. All participants were well informed about the study and provided informed written consent. Results were expressed in mean ± standard deviation.

### 2.2. Neuropsychological tests and analysis

Each participant underwent a set of neuropsychological tests using three questionnaires administered in a random order. These included the digit span (DS) Forward/Backward (DSF/DSB), the Verbal Fluency Test (VFT) of vegetables and fruits (VFT1) and the VFT of animals (VFT2), and the Symbol Digit Modalities Test (SDMT) written part and the SDMT oral part (Silva et al., 2018; Zhang et al., 2021). Univariate analysis of covariance (ANCOVA) with education level set as a covariate was used to compare among the three groups regarding their demographic and clinical characteristics as well as concerning (a) DSF and DSB, (b) VFT1 and VFT2, and (c) SDMT written and SDMT oral using SPSS software (Version 22.0).^1^ The Turkey *post-hoc* test was used to detect the difference between any two groups. A *p* < 0.05 was taken to infer statistical significance.

### 2.3. ERP task

#### 2.3.1. Task design

Four tasks were administered, including the Ongoing Task, the PM Task, the Oddball Task, and the Go/Nogo Task. They were adapted from the arrow-and-color bar task of previous studies (Burgess et al., 2001; Gao et al., 2013). To differentiate among various cognitive components, the same PM cue of the PM task was used in the Oddball Task and the Go/Nogo Task albeit with different instructions. The Ongoing Task was used to measure basic cognitive function and reaction speed. The Oddball Task and the Go/Nogo Task were designed to separately measure attention/working memory and inhibitory function. Measuring these cognitive functions may help identify the components responsible for impaired PM performance in TLE patients.

The computer screen background was in black. A white arrow pointing to the left or right was horizontally placed in the center of the screen with two parallel bars of different colors horizontally positioned at equal distances above and below the arrow (Burgess et al., 2001). The colors of the bars were randomly chosen from standard red, green and blue. Two horizontally arranged keyboards were used in the tasks with the left one for the left index finger and the right one for the right index and middle fingers (Gao et al., 2013). The Ongoing Task was always performed first, and this was followed by the PM Task, the Oddball Task and the Go/Nogo Task in a random but counterbalanced sequence among all the participants.

**FIGURE 1:**
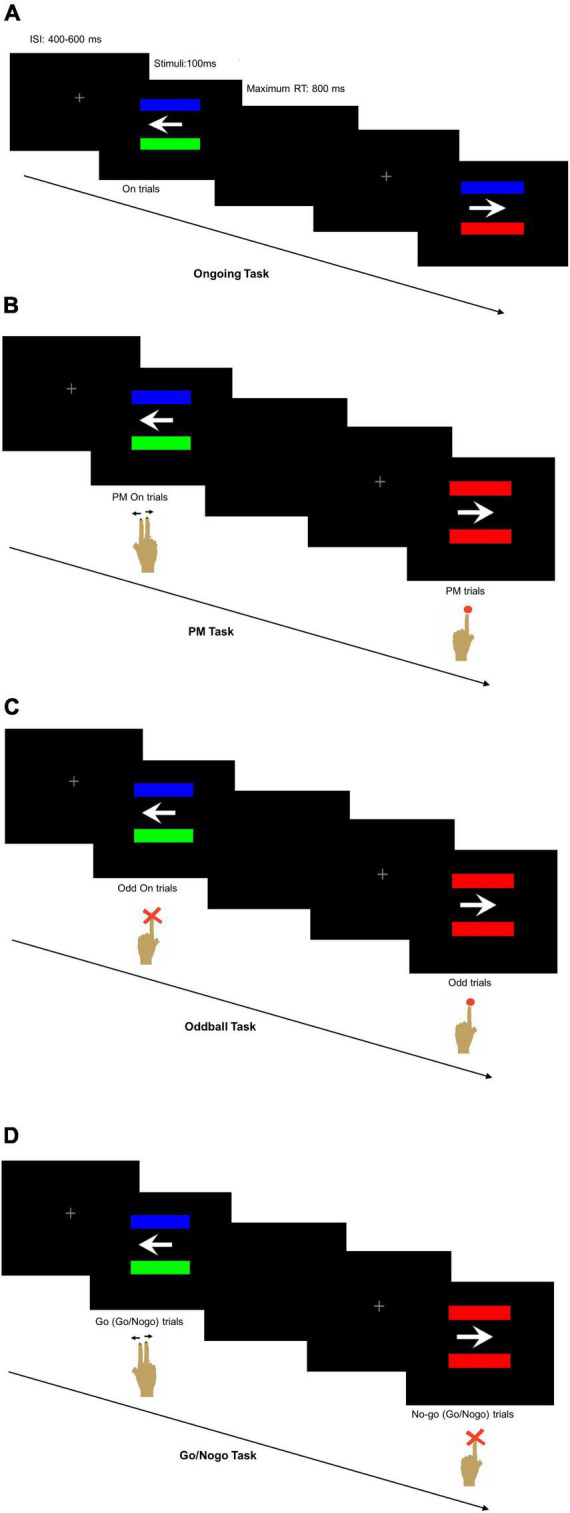
Computer screen appearance with two horizontally positioned parallel bars **(A)** of different colors in the Ongoing Task reminding the participant to press the right keyboard buttons according to the arrow direction, **(B)** either of different colors in the ongoing trials of prospective memory (PM) Task reminding the participant to press the right keyboard buttons according to the arrow direction or of same colors in the PM trials reminding the participant to press the left keyboard button, **(C)** either of different colors in the ongoing trials of Oddball Task reminding the participant not to press any button or of the same colors reminding the participant to press the left keyboard button, and **(D)** either of different colors reminding the participant to press the right keyboard buttons according to the arrow direction or of same colors reminding the participant not to press any button.

In the Ongoing Task, the participants were instructed to disregard the bar colors and press the right keyboard buttons with their right index finger for the left-pointing arrow and with their right middle finger for the right-pointing arrow (Figure 1A). The Ongoing Task had a total of 120 trials with 30 trials in each of the four sessions and a 5-s resting period between sessions.

In the PM Task, the participants were instructed to press the right keyboard buttons according to the arrow direction using the right index or middle finger like that of the Ongoing Task when the two bars were of different colors. Occasionally, the PM cues with two same-colored bars would appear, and the participants would disregard the arrow direction and press the left keyboard button using the left index finger (Figure 1B). The PM Task had a total of 240 trials with 30 trials in each of the eight sessions and a 5-s resting period between sessions. Six trials per session had the PM cues, making up a total of 48 randomly interpolated PM cues for all eight sessions of the PM Task. The first seven trials of each session would not contain the PM cues. The inter-stimulus interval (ISI) was randomly set at 400, 500, or 600 ms to avoid stimuli expectation.

In the Oddball Task, the participants were required to pay attention to the PM cues, disregard the arrow direction and respond to press the left keyboard button using their left index finger (Figure 1C). They would not press any button when the two bars were of different colors. In the Go/Nogo Task, the participants had to respond with their right index or middle finger according to the arrow direction when the bars were of different colors. They were instructed to disregard the arrow direction and refrain from pressing any button when seeing the PM cues (Figure 1D). The number of trials per session, the number of session per task, the number and random appearance of the PM cues, and the stimuli settings were similar to the PM Task. All the participants were given explicit instructions about the tasks so that they fully understood what to do for each task. In addition, the participants were given sufficient practice to ensure their accurate performance in each task.

#### 2.3.2. ERP data acquisition

Each participant would sit comfortably and calmly in an armchair inside a dimly lit room with a computer screen set about 60 cm in front of the face. A continuous recording of 128-channel scalp EEG (QuikCap, Compumedics Neuroscan, El Paso, TX, USA) and 4-channel horizontal and vertical electrooculography was made using Neuroscan 4.3 software (Compumedics, Neuroscan, El Paso, TX, USA). A band-pass filter of 0.01 to 100 Hz and a gain of 1,000 were used. Two reference electrodes were placed behind the left and right mastoids. The midline frontal electrode located on the EEG cap was the ground electrode. Electrode impedances were maintained below 30 kΩ (Sik et al., 2017).

#### 2.3.3. Behavioral data analysis

The behavioral data from the computer-based tests were recorded by the E-prime program.^2^ Trials with a reaction time more than 800 ms were excluded because these results reflected occasional inattention rather than poor test performance. All data sets were reassembled after primary data processing and then further analyzed using SPSS (Version 22.0). Missing data were excluded using listwise deletion. Repeated measures ANCOVA was used to test group differences in the four tasks with education level set as a covariate. The Turkey *post-hoc* test was also used. The strength of the linear relationship between the ERP behavioral data of each task and the results of psychological tests was evaluated using the Pearson correlation.

#### 2.3.4. ERP components of interest

Prospective positivity component: This component is an EPR indicator of switching from an ongoing to PM activity (Bisiacchi et al., 2009; West, 2011). Task switching is essential for PM performance in daily living (Costa et al., 2015; Faraut et al., 2021). PM deficits has been reported to adversely affect the recall of information about COVID pandemic (Aizpurua et al., 2021). The prospective positivity is related to the inhibition of the ongoing activity upon detection of a PM cue and the switching process (Joly-Burra et al., 2018).

P300 component: Oddball stimuli would generate a positive ERP wave at around 300 ms, which is the P300 component. P300 was calculated and measured in the time window of 300–600 ms after the stimuli, which is related to attention and working memory (Yao et al., 2014; Gregory et al., 2015). P300 may be generated from the functional interaction between the frontal lobe and hippocampus/temporal-parietal lobes (Huang et al., 2015). Utilizing a deviant oddball experiment, TLE patients have been found to exhibit reduced P300 amplitudes in the temporal region and, to a lesser extent, in the frontal region (Bocquillon et al., 2011; Artemiadis et al., 2014; Mukheem Mudabbir et al., 2021).

P3 component: It was measured in the Go/Nogo Task. P3 generally represents the inhibition process, which is another essential aspect of PM performance to suppress/delay a response or interrupt an activity and avoid interference (Johnstone et al., 2005). It is necessary for PM performance because participants often need to inhibit an ongoing task and switch to the PM task at the appropriate time (Schnitzspahn et al., 2013). The P3 component, an ERP marker associated with inhibition process (Polich, 2007), has been widely studied in neurological diseases such as schizophrenia (Farzan et al., 2010), attention-deficit hyperactivity disorder (Cubillo et al., 2010), and idiopathic generalized epilepsy (Chowdhury et al., 2014). Neuroimaging studies have shown evidence of prefrontal cortex involvement in the inhibitory response (Apsvalka et al., 2022; De Vis et al., 2022), with several types of inhibition mediated by other different cortical areas (Bokura et al., 2001; Ridderinkhof et al., 2004).

### 2.4. ERP data analysis

Event-related potential data were analyzed using Neuroscan 4.3 Software (Neurosoft, Inc., Sterling, VA, USA). The raw EEG data were filtered off-line with a zero phase-shift digital filter and a 0.1 to 30 Hz bandpass. Eye blink artifacts were mathematically corrected by the ocular reduction function of the software. EEG data were segmented into 800 ms epochs with a 100 ms pre-stimulus baseline according to the event markers. Time point zero indicated the start of the visual stimulus. Segments exceeding 100 μV were automatically discarded. Individual epochs were normalized relative to a 100 ms pre-stimulus baseline, and the calculated linear trend was removed across the entire epoch according to the pre-stimulus baseline. Same types of stimuli for trials of different tasks were averaged across all sessions to generate the group ERP. ERP components of interest, including the prospective positivity component, P300 component and P3 component, were identified (Schmiedt-Fehr and Basar-Eroglu, 2011; Cruz et al., 2016; Sowndhararajan et al., 2018). The mean amplitudes were calculated for selected ERP components with latencies from 400 to 700 ms in the PM Task, from 300 to 600 ms in the Oddball Task and from 350 to 600 ms in the Go/Nogo Task.

Scalp maps were obtained using the software to illustrate different ERP components, i.e., prospective positivity component in PM trials, P300 component in the Oddball trials, and P3 component in the nogo trials. Afterward, the mean amplitudes were compared among the HEA, WEL, and REF groups using ANCOVA with education level set as a covariate (SPSS Version 22.0). The Turkey *post-hoc* test was also used.

## 3. Results

### 3.1. Demographics of the participants

Sixty-four subjects aged 24–61 years participated in this study. The REF group had 20 patients (43.9 ± 11.6 years; 8 with left-sided TLE), and the WEL group had 18 patients (48.0 ± 12.0 years; 10 with left-sided TLE). Patients of the REF group had 16.0 ± 20.8 simple partial seizures and/or partial seizures with generalization in the past 6 months, whereas patients of the WEL group had no seizure within the same period. The HEA group had 26 subjects (43.0 ± 12.4 years). Years of education were not comparable among the groups with the HEA group having longer years of education than the two TLE groups but having no difference between REF and WEL groups. There were more left-sided or bilateral epileptic foci in the WEL group whilst the REF group had more right-sided epileptic foci. There were no significant differences among the groups in age, sex, epilepsy duration, age of onset of epilepsy, MRI/EEG/other evidence, and BDI scores (Table 1). The current drug treatment for each patient in the two TLE groups is listed in Supplementary Table 1.

**TABLE 1:** Demographic and clinical characteristics of the three groups of participants.

	HEA group (*n* = 26)	WEL group (*n* = 18)	REF group (*n* = 20)	*P*-value*
Age, years	43.0 ± 12.4	48.0 ± 12.0	43.9 ± 11.6	0.854
Sex, male/female	13/13	11/7	11/9	0.752
Education, years	15.8 ± 3.5	12.0 ± 4.9	11.6 ± 4.0	0.568
Epilepsy duration, years	NA	16.4 ± 7.6	24.2 ± 9.2	0.004
Onset age, years	NA	30.8 ± 14.9	19.0 ± 12.1	0.600
Location, left/right/bilateral	NA	10/2/5	8/11/1	0.009
Evidence, MRI/EEG/others	NA	14/12/0	17/12/3	0.084
Becker’s Depression Inventory	6.9 ± 4.3	8.3 ± 6.1	10.0 ± 5.0	0.178

HEA, healthy group; WEL, well-controlled; REF, refractory. *ANOVA or independent *t*-test.

### 3.2. Neuropsychological tests

After controlling for education level, ANCOVA revealed a significant difference in the DSF scores [*F*(2,61) = 3.756, *p* = 0.029] among the groups (Table 2). *Post-hoc* test showed that the HEA group had significantly higher scores than the WEL group (*p* = 0.025) and the REF group (*p* = 0.024). There were also significant intergroup differences in the VFT1 scores [*F*(2,61) = 3.360, *p* = 0.040] and the VFT2 scores [*F*(2,61) = 3.950, *p* = 0.019]. *Post-hoc* test showed that the HEA group scored higher than the REF group in VFT1 (*p* < 0.01) and VFT2 (*p* = 0.033). In addition, the REF group scored lower than the WEL group in VFT2 (*p* < 0.01; Table 2). There was a trend for intergroup difference in SDMT oral scores because of the low score in REF group when compared to the HEA group.

**TABLE 2:** Neuropsychological tests.

	HEA group	WEL group	REF group	ANCOVA *P*-value		*Post hoc*	
					HEA vs. WEL	HEA vs. REF	WEL vs. REF
DSF	9.7 ± 1.1	8.3 ± 1.2	8.1 ± 1.8	0.029*	0.025*	0.024*	0.960
DSB	7.7 ± 1.9	6.3 ± 1.7	6.0 ± 2.1	0.528	0.320	0.345	0.968
VFT1	21.2 ± 3.3	17.3 ± 9.6	14.2 ± 4.3	0.040*	0.244	0.006**	0.162
VFT2	21.8 ± 4.0	20.0 ± 10.8	14.3 ± 4.2	0.019*	0.775	0.033*	0.008**
SDMT written	59.0 ± 10.6	52.7 ± 17.3	47.0 ± 13.6	0.386	0.761	0.197	0.313
SDMT oral	66.9 ± 12.2	58.4 ± 16.0	51.3 ± 15.3	0.093	0.383	0.038*	0.197

DSB, digit span backward; DSF, digit span forward; HEA, healthy; WEL, well-controlled; REF, refractory; SDMT, symbol digit modalities test; VFT1, verbal fluency test1 (fruits); VFT2, verbal fluency test2 (animals). ANCOVA: *p < 0.05; **p < 0.01.

### 3.3. Behavioral results from ERP tasks

Across all the groups, the participants tended to perform the ongoing trials faster than other trials with the shortest reaction time (Supplementary Figure 1). The reaction time tended to be progressively longer in odd trials of the Oddball Task, nogo trials of the Go/Nogo Task, ongoing trials of the PM Task and, finally, PM trials of the PM Task. Owing to the small sample sizes with relatively large variations, the trends were not significant statistically.

Table 3 summarizes the accuracy and reaction time of the three groups during different ERP tasks. The REF group tended to be less accurate than the other two groups in all except ongoing trials of the Oddball Task. Repeated measures ANCOVA with education level as the covariate revealed that the differences in accuracy were not significant except for doing the ongoing trials in Go/Nogo Task (*p* = 0.015) and PM task (*p* < 0.05) with the REF group being worse than HEA group as well as responding to PM cues in the PM Task (*p* < 0.001) with the REF group being worse than the other two groups and the WEL group worse than the HEA group. The HEA group tended to have a shorter reaction time than the TLE groups whilst the REF group tended to have a longer reaction time than the other two groups (Table 3). Nevertheless, the differences were not significant according to ANCOVA.

**TABLE 3:** Behavioral results from ERP tasks.

	HEA group	WEL group	REF group	ANCOVA *P*-value	*Post hoc*		
					HEA vs. WEL	HEA vs. REF	WEL vs. REF
**Accuracy (%)**							
**On**	94.8 ± 8.9	93.6 ± 9.4	89.4 ± 10.9	0.165	0.916	0.154	0.378
**Odd on**	99.9 ± 0.3	99.6 ± 0.7	99.6 ± 0.6	0.163	0.179	0.344	0.913
**Odd**	96.7 ± 7.3	96.5 ± 4.6	94.5 ± 12.0	0.65	0.996	0.657	0.752
**Go/Nogo on**	96.4 ± 8.0	93.6 ± 10.4	82.8 ± 24.8	0.015*	0.832	0.014*	0.095
**Go/Nogo**	90.2 ± 7.0	92.1 ± 5.7	88.8 ± 5.8	0.296	0.592	0.761	0.266
**PM on**	89.1 ± 4.9	87.2 ± 6.2	85.0 ± 5.0	0.043*	0.501	0.033*	0.406
**PM**	90.9 ± 6.2	85.5 ± 6.3	79.7 ± 7.1	<0.001**	0.024*	<0.001**	0.023*
**Reaction Time (ms)**							
**On**	435.7 ± 55.3	444.4 ± 73.2	459.2 ± 62.7	0.46	0.895	0.428	0.751
**Odd on**	NA	NA	NA	NA	NA	NA	NA
**Odd**	459.0 ± 49.6	455.9 ± 61.6	466.1 ± 52.6	0.833	0.981	0.898	0.831
**Go/Nogo on**	490.8 ± 50.5	495.8 ± 77.1	502.8 ± 45.3	0.786	0.956	0.767	0.928
**Go/Nogo**	NA	NA	NA	NA	NA	NA	NA
**PM on**	498.4 ± 52.9	511.2 ± 75.0	533.4 ± 42.3	0.128	0.746	0.108	0.463
**PM**	548.2 ± 66.0	561.0 ± 65.7	568.9 ± 45.1	0.504	0.768	0.484	0.914

ERP, event-related potentials; HEA, healthy; Go/Nogo, Nogo trials in the Go/Nogo Task; Go/Nogo on, ongoing trials in the Go/Nogo Task; NA, not available; Odd, oddball trials in the Oddball Task; Odd on, ongoing trials in the Oddball Task; On, ongoing trials in Ongoing Task; PM, prospective memory (PM) trials in the PM Task; PM on, ongoing trials in the PM Task; WEL, well-controlled; REF, refractory. ANCOVA: *p < 0.05; **p < 0.01.

Supplementary Table 2 summarizes the Pearson correlation analysis results between the ERP behavioral data and neuropsychological tests of the questionnaires. Except for responding to PM cues of the Oddball Task and SDMT written scores in ongoing trials of the Go/Nogo Task, the accuracy of performing ongoing trials of the Ongoing Task, Go/Nogo Task and PM task as well as the accuracy of responding to PM cues of the PM Task were positively correlated with the results of neuropsychological tests (*p* < 0.05). The reaction time of performing ongong trials of the Ongoing Task was negatively correlated with the results of neuropsychological tests (*p* < 0.05; Supplementary Table 2). The reaction time of performing ongoing trials of the Go/Nogo Task was negatively correlated with the VFT1 and VFT2 scores (*p* < 0.01), and the reaction time of performing ongoing trials of the PM Task was negatively correlated with the DSF, VFT1 and VFT2 scores (*p* < 0.05).

### 3.4. ERP components of interest

#### 3.4.1. PM Task

**FIGURE 2:**
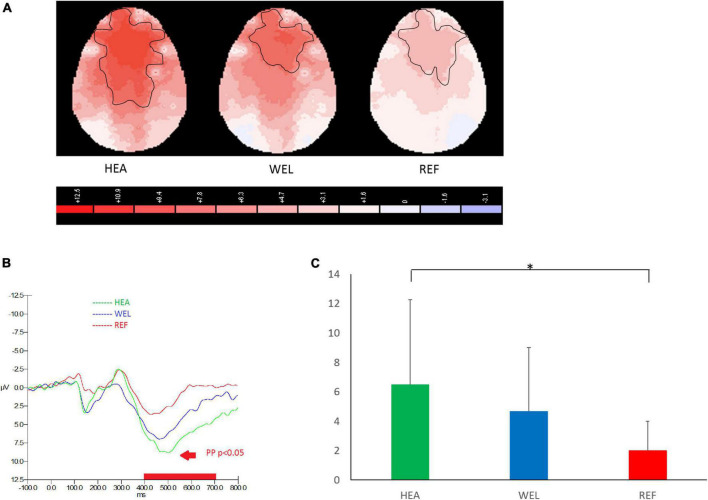
Prospective positivity (PP; 400–700 ms) during prospective memory (PM) trials of the PM Task with **(A)** 2-D scalp maps showing strongest and largest activations over the frontal, central and parietal sites in the healthy (HEA) controls, weakest and smallest activations in the refractory (REF) group of epilepsy patients and intermediate activations in the well-controlled (WEL) group, **(B)** grand average waveforms at Fz of the three groups, and **(C)** mean amplitudes at Fz of the three groups. Significant intergroup difference according to ANCOVA with education level set as the covariate. *p < 0.05 between the HEA and REF groups on the *post-hoc* test.

As an ERP indicator of responding to PM cues of the PM Task, prospective positivity between 400 and 700 ms was found over the frontal, central and parietal sites, and the outlined region in the scalp map represented the activated areas (Figure 2; see Supplementary Figure 2 for the grand average ERP waveforms). The mean amplitudes of prospective positivity were as follows: the HEA group, Fz 6.49 ± 5.79 μV, Cz 7.84 ± 6.22 μV, Pz 5.15 ± 5.00 μV; the WEL group, Fz 4.67 ± 4.36 μV, Cz 5.03 ± 5.87 μV, Pz 3.85 ± 4.83 μV; and the REF group, Fz 1.98 ± 2.01 μV, Cz 1.59 ± 1.85 μV, Pz 1.46 ± 1.91 μV. ANCOVA with education level set as the covariate revealed a significant intergroup difference in the frontal, central and parietal sites [e.g., Fz, *F*(2,61) = 4.054, *p* = 0.022] with the *post-hoc* tests showing a significant difference between the HEA and REF groups (*p* < 0.01) but not between the HEA and WEL groups (*p* = 0.287) and between the WEL and REF groups (*p* = 0.084).

#### 3.4.2. Oddball Task

**FIGURE 3:**
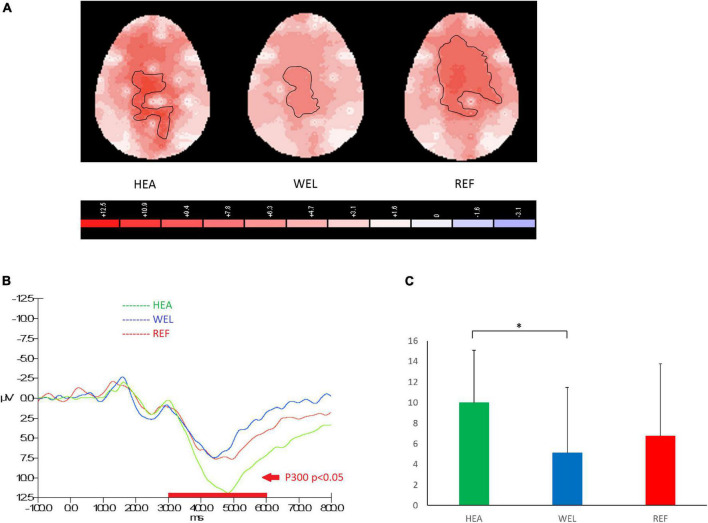
P300 component (300–600 ms) during oddball trials of the Oddball Task with **(A)** 2-D scalp maps showing stronger and larger activations over the central-parietal sites in the healthy (HEA) controls and refractory (REF) group of epilepsy patients but weaker and smaller activations in the well-controlled group, **(B)** grand average waveforms at Pz of the three groups, and **(C)** mean amplitudes at Pz of the three groups. Significant intergroup difference according to ANCOVA with education level set as the covariate. *p < 0.05 between the HEA and WEL groups on the *post-hoc* test.

The amplitude of P300 between 300 and 600 ms over the central-parietal sites in response to PM cues of the Oddball Task was used to measure attention and working memory, and the outlined region in the scalp map represented the activated areas (Figure 3; see Supplementary Figure 3 for the grand average ERP waveforms). The mean amplitudes of P300 were as follows: HEA group, Fz 6.64 ± 4.78 μV, Cz 10.97 ± 6.04 μV, Pz 9.98 ± 5.09 μV; WEL group, Fz 3.80 ± 5.77 μV, Cz 6.37 ± 7.75 μV, Pz 5.10 ± 6.37 μV; and the REF group, Fz 7.18 ± 6.39 μV, Cz 9.16 ± 8.25 μV, Pz 6.78 ± 6.99 μV. ANCOVA with education level set as the covariate revealed a significant intergroup difference in the central-parietal sites [e.g., Pz, *F*(2,61) = 3.185, *p* = 0.048] with the *post-hoc* tests showing a significant difference between the HEA and WEL groups (*p* < 0.05) but not between the HEA and REF groups and between the WEL (*p* > 0.05) and REF groups (*p* > 0.05).

#### 3.4.3. Go/Nogo Task

**FIGURE 4:**
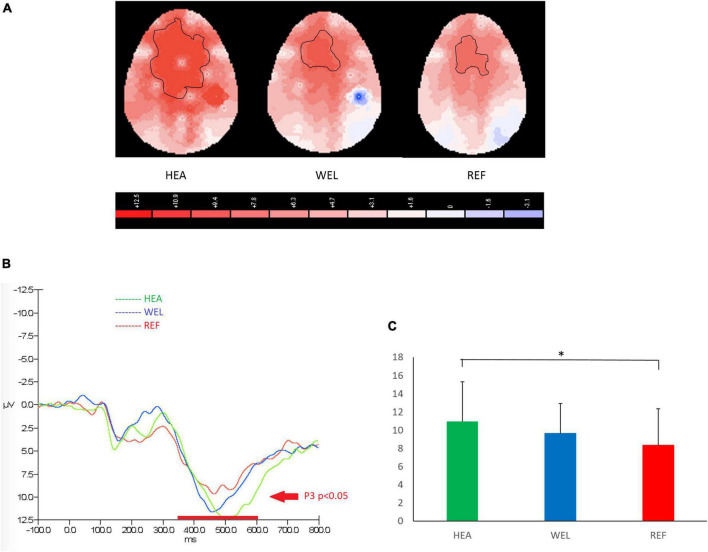
P3 (350–600 ms) during nogo trials of the Go/Nogo Task with **(A)** 2-D scalp maps showing strongest and largest activations over the frontal-central sites in the healthy (HEA) controls, weakest and smallest activations in the refractory (REF) group of epilepsy patients and intermediate activations in the well-controlled (WEL) group, **(B)** grand average waveforms at Fz of the three groups, and **(C)** mean amplitudes at Fz of the three groups. Significant intergroup difference according to ANCOVA with education level set as the covariate. *p < 0.05 between the HEA and REF groups on the *post-hoc* test.

A P3 component between 350 and 600 ms and peaked at around 500 ms was observed over the frontal-central sites to indicate inhibition in response to PM cues of Go/Nogo Task, and the outlined region in the scalp map represented the activated areas (Figure 4; see Supplementary Figure 4 for the grand average ERP waveforms). The mean amplitudes of Nogo P3 were as follows: the HEA group, Fz 10.94 ± 4.35 μV, Cz 10.40 ± 6.87 μV, Pz 7.85 ± 3.33 μV; the WEL group, Fz 9.63 ± 3.29 μV, Cz 9.88 ± 3.99 μV, Pz 4.78 ± 4.42 μV; and the REF group, Fz 8.34 ± 3.98 μV, Cz 8.67 ± 5.30 μV, Pz 5.29 ± 4.44 μV. ANCOVA with education level set as the covariate revealed a significant intergroup difference in the frontal-central sites [e.g., Fz, *F*(2,61) = 3.786, *p* = 0.033] with the *post-hoc* tests showing a significant difference between the HEA and REF groups (*p* = 0.04) but not between the HEA and WEL groups (*p* > 0.05) and between the WEL and REF groups (*p* > 0.05).

## 4. Discussion

Medial temporal lobe damage is associated with PM impairment to indicate the role of episodic memory (Burgess et al., 2007; Kliegel et al., 2008). TLE patients have temporal lobe dysfunctions, including impairment in episodic memory and PM (Jain et al., 2018; Li et al., 2022). Successful PM performance requires not only memory function but also attention, inhibition, and other execution functions of the frontal lobe. Indeed neurological and psychiatric patients with frontal neural circuitry damage frequently exhibit PM deficits (Kliegel et al., 2008). Thus, PM deficits in TLE patients warrant further investigations especially on its relatively less well studied prospective component of PM. In this first ERP study on PM impairment in TLE patients, the REF group have more left temporal lobe abnormalities. Patients with mesial temporal sclerosis on the left side have greater PM impairment than those with lesion on the right side (Adda et al., 2008).

The present results in DS, VFT and SDMT indicated impaired working memory, verbal function, and information processing speed, respectively, in TLE patients. The impairments are primarily found in the REF group of patients whereas the WEL group’s worse DSF score indicated impaired working memory. VFT2 score was lower in the REF group of patients when compared to the WEL group to reveal an impairment of semantic fluency which is a frontotemporal lobe function (Troyer et al., 1998; Lepow et al., 2010).

Recent literature has reported generalized cognitive impairment, particularly executive function and processing speed, in TLE to implicate a more extensive network dysfunction beyond the frontotemporal areas responsible for working memory and executive function (Oyegbile et al., 2018). Our neuropsychological results are in line with global cognitive impairment in TLE patients. Further correlation analysis has revealed potential association between PM task accuracy and working memory, semantic fluency, and processing speed. Our earlier study showed that declined processing speed in verbal and non-verbal tasks could explain the cognitive deficits in abnormal aging (dementia) and normal aging (Gao et al., 2013). As a network disease, therefore, it is plausible that TLE patients have various cognitive deficits.

Various cognitive resources such as attention, inhibition, and execution are required in successful PM performance. Behavioral results of our ERP experiments have revealed a longer response time in the PM Task when compared to other tasks in both groups of TLE patients and HEA group of controls. The REF group of patients tended to have the longest reaction time during the PM Task but the intergroup differences are not significant. Worst PM Task accuracy in the REF group of patients would indicate their impaired PM function. ERP studies on the PM Task could elucidate the potential neural mechanisms underlying PM dysfunction (Trimmel et al., 2017). In addition, we employed the Oddball Task and Go/Nogo Task to distinguish between attention/executive function and inhibitory functions which are critical in successful PM performance (McDaniel and Einstein, 2007, 2011).

Prospective positivity was clearly observed in all three groups during the PM Task with the lowest amplitude in the REF group of patients which is significantly reduced when compared to the HEL group of controls. As a key EPR indicator of PM function, prospective positivity is broadly seen over the central, parietal, and occipital regions of the scalp (West and Krompinger, 2005). In the present study, prospective positivity is mainly observed in the frontocentral brain regions responsible for task configuration, coordination, and task switching (Bisiacchi et al., 2009; Cruz et al., 2016; Mitra et al., 2022). Our findings suggest a role of prospective positivity in switching between ongoing activities and the PM Task. This is consistent with reported findings (Cona et al., 2014; Cejudo et al., 2022; Crook-Rumsey et al., 2022). The late positive complex has been postulated to reflect different cognitive processes, depending on whether the instructions would involve a dual-task or task-switch approach (Bisiacchi et al., 2009).

It has been proposed that patients with refractory TLE may have disrupted brain networks especially the fronto-central-temporal network (Lin et al., 2020). Other investigators and theorists have stressed on an interplay between the medial temporal lobe memory system and the frontal executive system in supporting PM function (Palmer and McDonald, 2000; Zimmer et al., 2001). PM studies using both functional neuroimaging and neuropsychological techniques including positron emission tomography, functional MRI (fMRI) and magnetoencephalography have revealed consistent activations in the lateral and orbital prefrontal cortices (Burgess et al., 2007, 2008) as well as the medial temporal lobe (Martin et al., 2007) during PM activities. Depending on the nature of different PM activities (Luck, 2014), prospective positivity is consisted of a variety of ERP components, including the P3b component (West and Wymbs, 2004; West et al., 2006), the late positive complex associated with task configuration (Bisiacchi et al., 2009) and the old-new recognition effect (West and Krompinger, 2005).

P300 component of the EPR response during the Oddball Task was mainly seen in central-parietal leads (Chen et al., 2001; Rocha et al., 2010). P300 components of lower amplitudes and longer latencies have been reported in other studies (Grunwald et al., 1999; Rocha et al., 2010). Two recent studies have reported decreased frontoparietal connectivity in TLE patients (Riley et al., 2010; Liao et al., 2011). TLE patients with unilateral hippocampal sclerosis have impaired working memory, patients with left or right-sided TLE have decreased right superior parietal lobe activation during working memory tasks, and progressive hippocampal deactivation is observed upon increasing task demands (Stretton and Thompson, 2012; Duarte et al., 2014). On the other hand, several imaging studies have also reported hippocampal activation during working memory tasks involving encoding, maintenance and retrieval processes (Axmacher et al., 2007; Mainy et al., 2007; Schon et al., 2009). Taken together, temporal lobe dysfunction plays a critical role of in working memory impairment, and the parietal lobe may also be involved. The propagation of epileptic activity from the epileptogenic zone to the eloquent cortex may lead to specific cognitive dysfunction (Hermann et al., 1988).

In the present study, P300 amplitudes in the Oddball Task were significantly lower in the WEL group but not REF group of patients when compared to the HEA group of controls, revealing impaired attention/working memory in stable but not unstable TLE patients. Our P300 amplitude results of WEL group but not REF group are in agreement with the literature (Schomaker et al., 2021). P300 amplitude has also been found to reflect treatment effectiveness (Sun et al., 2008). For example, increased P300 amplitude at the parietal midline (Pz) electrode was observed when the epilepsy responded well to vagus nerve stimulation therapy (De Taeye et al., 2014; Wostyn et al., 2017). Further studies are needed to explain our P300 amplitude findings in REF group. A plausible explanation is that there are more left-sided epileptic foci in the WEL group whilst patients of the REF group have more right-sided epileptic foci. In TLE patients with mesial temporal sclerosis, reduced P300 is seen especially in those with left-sided sclerosis (Grunwald et al., 1999). Another ERP study has reported that patients with left-sided mesial temporal sclerosis had P300 with lower amplitude and longer latency than controls, mainly at the central C3 and C4 regions (Rocha et al., 2010). The present study did not find any significant difference in P300 latency. Some ERP studies on epilepsy patients have reported longer latency of P300 (Naganuma et al., 1997; Wu et al., 1997; Sowndhararajan et al., 2018). Nevertheless, other studies have reported no change in P300 latency in epilepsy patients and no effect from epilepsy treatment (Sun et al., 2008; Boscariol et al., 2015).

Nogo P3 is linked to inhibitory neural activity in the frontal lobe. To our knowledge, there is no published ERP study on inhibitory function in TLE patients; P3 component of the EPR response during the Nogo Task was mainly seen in frontocentral leads. An important role of frontocentral sites in inhibition is in line with the literature (Aron et al., 2004; Swick et al., 2008). On the other hand, there is a good correlation between orbitofrontal cortex activity on fMRI and behavioral performance in Go/Nogo tasks (Criaud and Boulinguez, 2013). Previous EPR studies have localized the Nogo P3 to the left orbitofrontal cortex (Falkenstein et al., 1999) or the left lateral orbitofrontal area (Bokura et al., 2001). Patients with frontal cortex damage have abnormal social behavior, including inappropriate activities and disinhibited behavior (Deets et al., 1970; Kratsman et al., 2016), and they also have impaired PM function (Burgess et al., 2008).

Nogo P3 amplitudes were significantly lower in the REF group but not WEL group of patients when compared to the HEA group of controls, revealing impaired inhibition in unstable but not stable TLE patients. Taken together with the amplitudes of prospective positivity and P300 components, the neuromechanism for PM impairment in REF group of patients may involve impaired task switching and inhibition rather than attention deficit. Despite the attention deficit in the WEL group of patients, PM impairment was not observed when their frontal inhibitory function was unaffected. In other words, the present findings of double dissociation in EPR components suggest that inhibition dysfunction is a more important mechanism than attention deficit in causing PM deficit. It is speculated that frequent seizures in REF group of patients may spread to affect other brain regions, such as the frontal lobe, anterior cingulate cortex and thalamus, resulting in compromised inhibitory process (Akinci et al., 2023).

There has been a growing body of research on the neuropsychological deficits and large-scale disorganization of memory and executive function in TLE patients (Hermann and Seidenberg, 2007; Reyes et al., 2019; Caciagli et al., 2023). The present results support the notion that epilepsy is a disorder of brain network and that refractory TLE is associated with significant cognitive impairments. More research on the underlying neural mechanisms of cognitive deficits is needed to improve our diagnosis, treatment and management. Given the importance of PM performance in daily living and the dependence of PM on various cognitive components, disrupted neural network information processing would cause PM impairment in TLE and other epilepsies. PM deficit can be an important biomarker of severity in TLE. As such, research on antiepileptic drugs should go beyond seizure control and include cognitive functions and PM performance as potential benefits or adverse effects (Barr, 2002; Mattson, 2004).

This study has two limitations. First, the number of left- and right-sided lesions was imbalanced in the two groups of TLE patients. This important confounder should be addressed in future studies to better understand the role of lateralization in PM deficits of TLE patients. Second, the Pearson correlation analysis results between the ERP behavioral data and neuropsychological tests of the questionnaires are both exploratory and preliminary findings. Larger sample sizes with increased statistical power are needed to study on the correlation between ERP components and neuropsychological tests.

In summary, three ERP components were studied in TLE patients and healthy subjects during the PM Task, Oddball Task and Go/Nogo Task with ERP results correlated with neuropsychological tests and behavioral data to delineate the relevance of attention and inhibition in PM function. TLE patients were separated into two groups according to their seizure control. Impaired PM function in refractory TLE patients may be attributed to their impaired inhibition over frontal-central sites since well-controlled TLE patients had no PM deficit despite their deficit in attention. Our findings on this double dissociation suggest that the adverse effects of TLE on PM function may be more dependent on inhibitory dysfunction than attention dysfunction. Nevertheless, there are other factors for PM impairment in refractory TLE patients, including antiepileptic drugs, depression, and sociological stigma. Further studies are needed. The current finding of PM deficits in refractory TLE patients have clinical implications in their daily living and neuropsychological rehabilitation.

## Data availability statement

The raw data supporting the conclusions of this article will be made available by the authors, without undue reservation.

## Ethics statement

The studies involving human participants were reviewed and approved by the Institutional Review Board of the University of Hong Kong/Hospital Authority Hong Kong West Cluster (HKU/HA HKW IRB). The patients/participants provided their written informed consent to participate in this study.

## Author contributions

HY and JG conducted the experiment and drafted the manuscript. RS-KC designed the experiment. WM recruited the patients and revised the manuscript. T-QT reviewed the statistical analyses and revised the manuscript. RC supervised and coordinated the study and critically revised manuscript. All authors contributed to the article and approved the submitted version.

## Footnotes

1
www.ibm.com/products/spss-statistics

2
https://pstnet.com/products/e-prime/
