# Supplementary material for: Inhibitory dysfunction may cause prospective memory impairment in temporal lobe epilepsy (TLE) patients: an event-related potential study
Source: Front Hum Neurosci. 2023 Jul 26;17:1006744. doi: 10.3389/fnhum.2023.1006744 (PMC10410078; doi:10.3389/fnhum.2023.1006744)

**Supplementary Table 1. Current drug treatment for each patient**

| **WEL** | | **REF** | |
| --- | --- | --- | --- |
| **Patient** | **AED** | **Patient** | **AED** |
| 1 | LTG | 1 | LVT |
| 2 | OXC | 2 | LTG+LVT+LCM |
| 3 | LVT | 3 | LTG+VPA |
| 4 | VPA | 4 | CBZ |
| 5 | NA | 5 | CBZ+LCM |
| 6 | LVT | 6 | OXC+LVT+VPA |
| 7 | CBZ | 7 | VPA+LVT+PHT |
| 8 | PB | 8 | LTG+OXC+BDZ |
| 9 | OXC | 9 | LVT+OXC+BDZ |
| 10 | VPA | 10 | LTG+LVT |
| 11 | VPA | 11 | VPA+BDZ |
| 12 | PHT | 12 | CBZ+LTG |
| 13 | VPA | 13 | LVT+OXC |
| 14 | CBZ | 14 | LEV+PB+BDZ+LCM |
| 15 | NA | 15 | CBZ+LVT+PHT+BDZ |
| 16 | NA | 16 | LVT+CBZ+BDZ |
| 17 | LVT | 17 | LCM+OXC+LVT+BDZ |
| 18 | VPA | 18 | LVT+LTG+BDZ |
|  |  | 19 | LVT+CBZ+BDZ |
|  |  | 20 | CBZ |

AED, antiepileptic drugs; BDZ, benzodiazepine; CBZ, carbamazepine; LCM, lacosamide; LTG, lamotrigine; LVT, levetiracetam; NA, not available; OXC, oxcarbazepine; PB, phenobarbital; PHT, phenytoin; REF, refractory; VPA, valproate; WEL, well-controlled

**Supplementary Table 2. Correlation between the ERP behavioral tests and neuropsychological tests**

|  |  | **DSF** | **DSB** | **VFT1** | **VFT2** | **SDMT written** | **SDMT oral** |
| --- | --- | --- | --- | --- | --- | --- | --- |
| **Accuracy** | On | 0.485** | 0.460** | 0.470** | 0.461** | 0.338** | 0.388** |
|  | Odd | 0.192 | 0.132 | 0.170 | 0.148 | 0.196 | 0.120 |
|  | Go/Nogo on | 0.489** | 0.476** | 0.474** | 0.481** | 0.371** | 0.325** |
|  | PM on | 0.469** | 0.364** | 0.598** | 0.580** | 0.130 | 0.279* |
|  | PM | 0.469** | 0.415** | 0.410** | 0.390** | 0.423** | 0.345** |
| **Reaction Time** | On | -0.338** | -0.339** | -0.508** | -0.551** | -0.165** | -0.145** |
|  | Odd | 0.059 | -0.026 | -0.180 | -0.197 | -0.231 | -0.140 |
|  | Go/Nogo on | -0.175 | -0.090 | -0.408** | -0.425** | -0.090 | -0.085 |
|  | PM on | -0.260* | -0.245 | -0.466** | -0.483** | -0.131 | -0.130 |
|  | PM | 0.003 | -0.070 | -0.198 | -0.197 | -0.049 | -0.134 |

DSB, digit span backward; DSF, digit span forward; ERP, event-related potentials; Go/Nogo on, ongoing trials in the Go/Nogo Task; Odd, oddball trials in the Oddball Task; On, ongoing trials in Ongoing Task; PM, prospective memory (PM) trials in the PM Task; PM on, ongoing trials in the PM Task; SDMT, symbol digit modalities test; VFT1, verbal fluency test1 (fruits); VFT2, verbal fluency test2 (animals)

*, p<0.05; **, p<0.01

Supplementary Figure 1. Reaction time (ms) during different trials of the ERP tasks.

Go (Go/Nogo), nogo trials; HEA, healthy; Odd, oddball trials; On, ongoing trials; PM, ongoing trials in Prospective Memory (PM) Task; PM On, PM trials; REF, refractory; WEL, well-controlled.


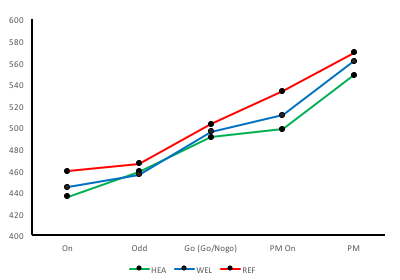


Supplementary Figure 2. The grand average ERP waveforms of prospective positivity (PP) during ongoing trials and prospective memory (PM) trials of the PM Task in three representative channels (Fz, Cz, Pz) of the three groups of participants: healthy (HEA) controls; well-controlled (WEL) epilepsy patients; and refractory (REF) epilepsy patients.


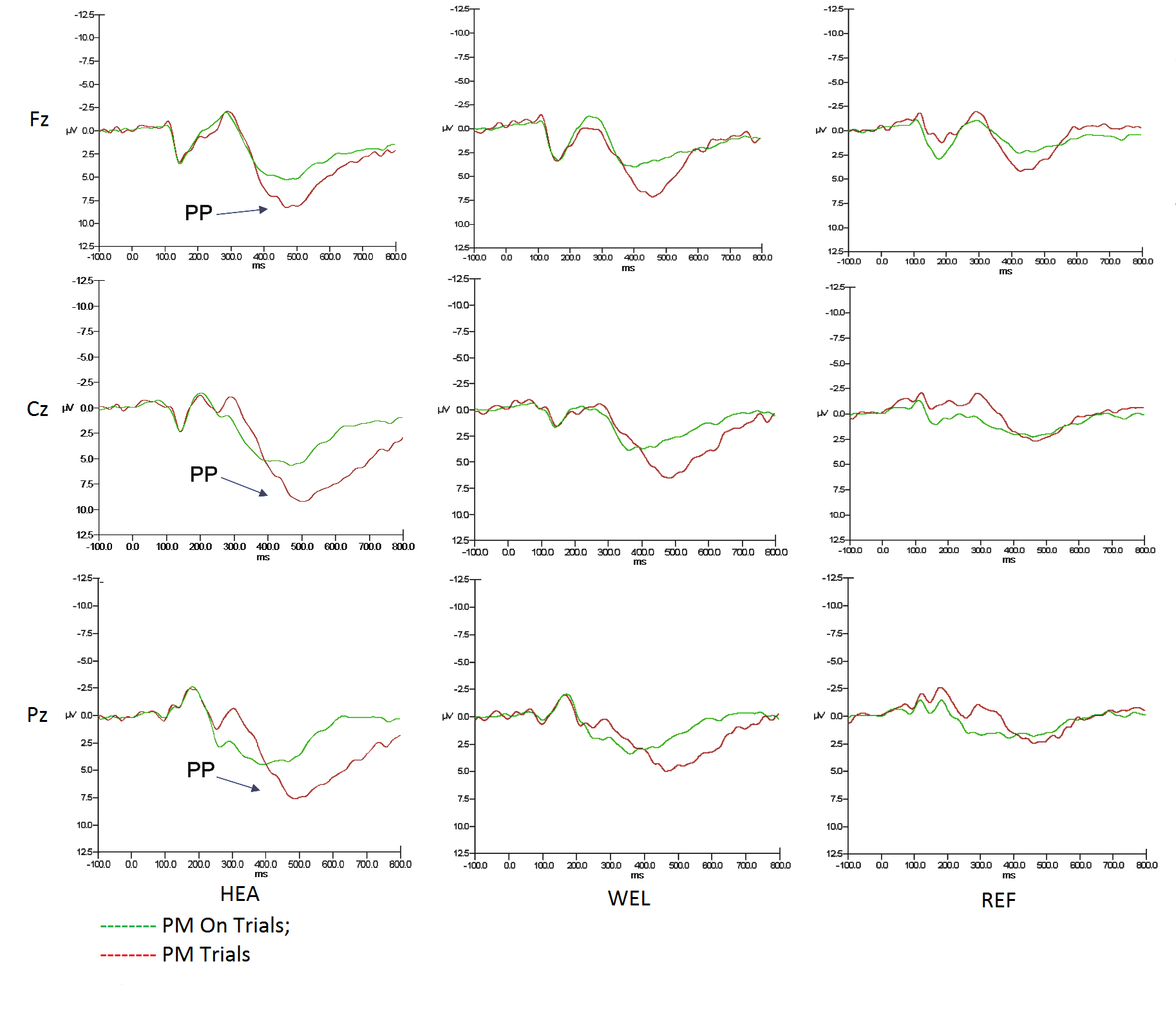


Supplementary Figure 3. The grand average ERP waveforms of P300 during ongoing trials and oddball trials of the Oddball Task in three representative channels (Fz, Cz, Pz) of the three groups of participants: healthy (HEA) controls; well-controlled (WEL) epilepsy patients; and refractory (REF) epilepsy patients.


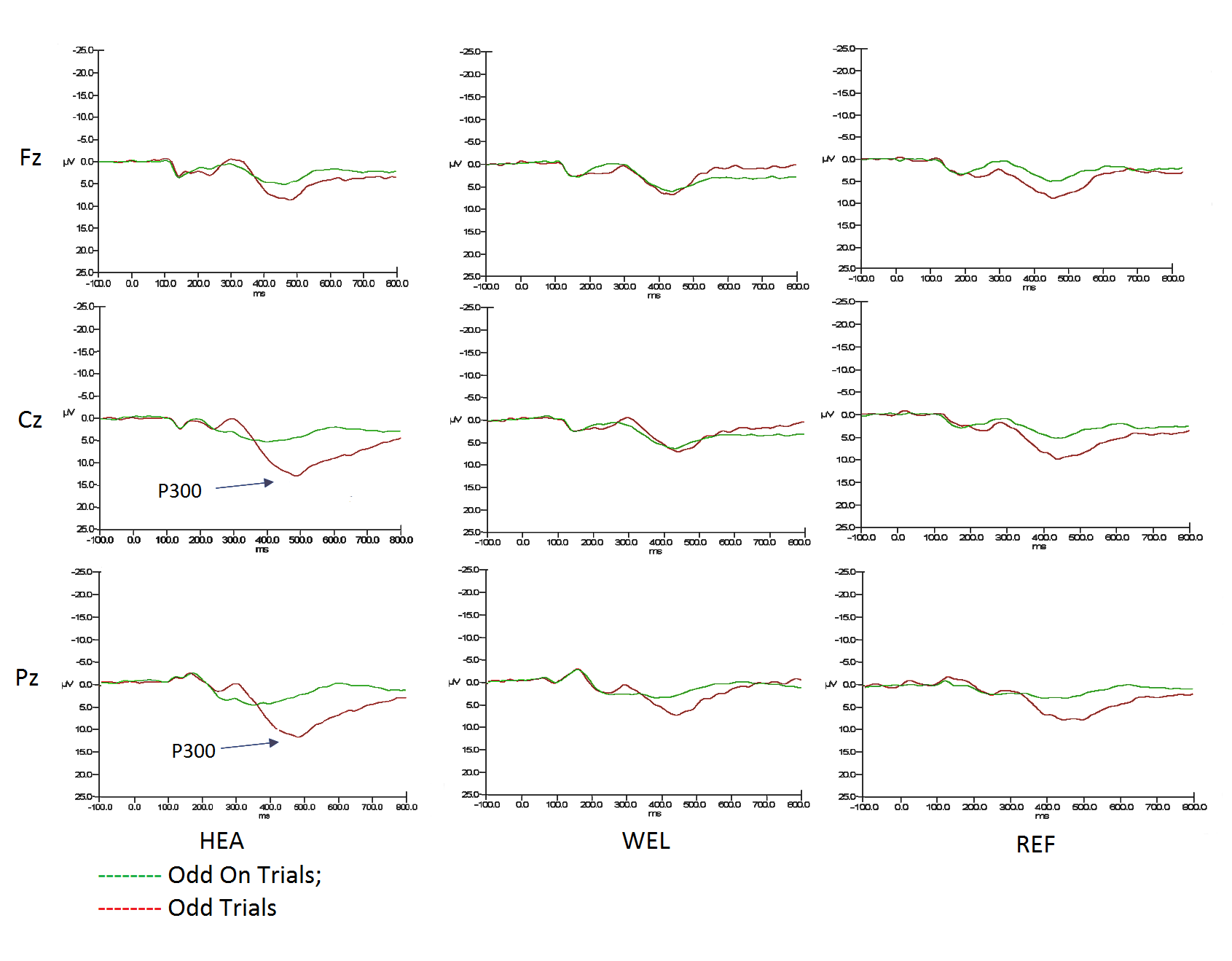


Supplementary Figure 4. The grand average ERP waveforms of P3 during ongoing (go) trials and nogo trials of the Go/Nogo Task in three representative channels (Fz, Cz, Pz) of the three groups of participants: healthy (HEA) controls; well-controlled (WEL) group of epilepsy patients; and refractory (REF) group of epilepsy patients.


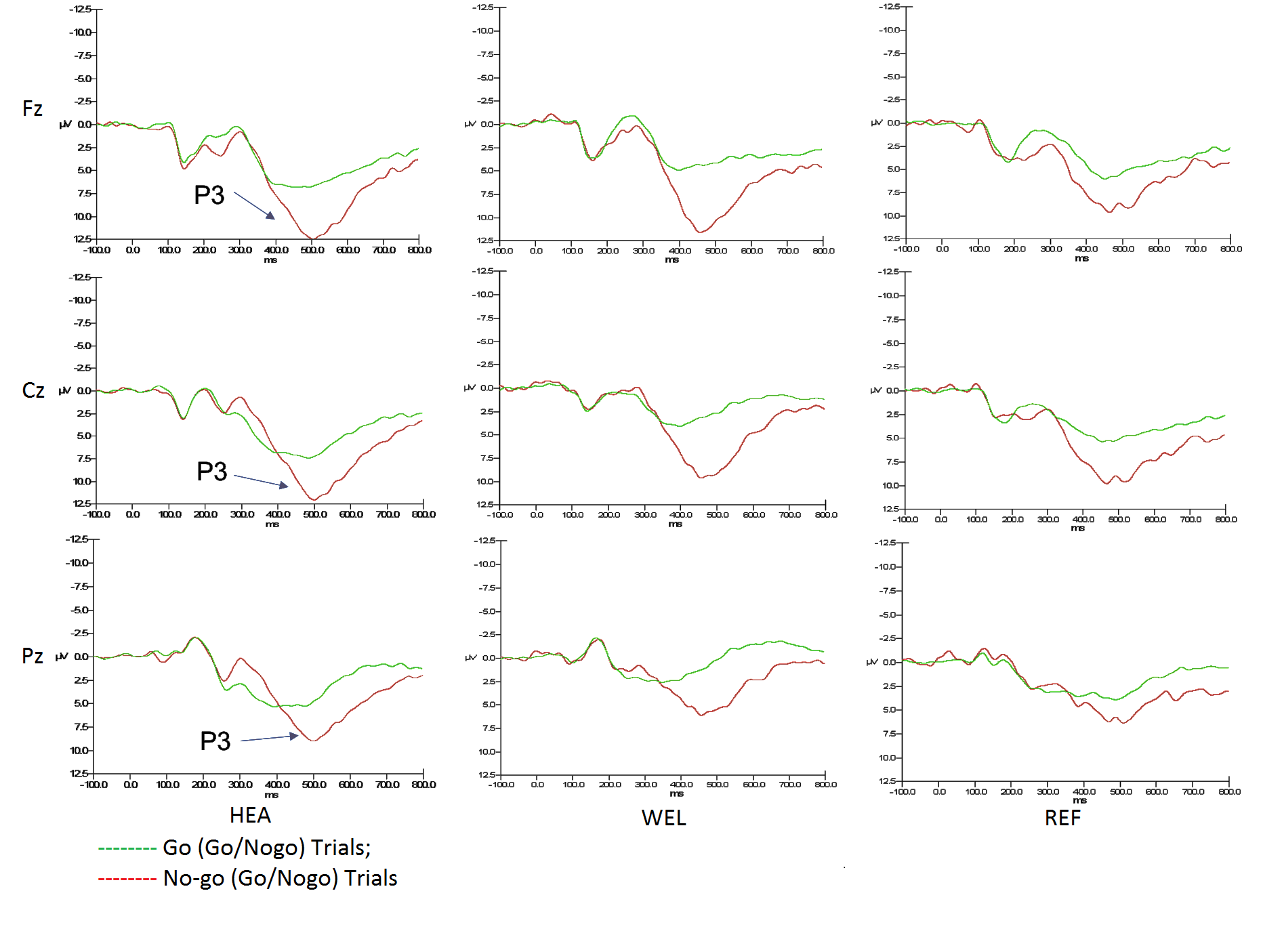

Supplement: Supplementary file 1 [file Data_Sheet_1.docx]
